# A community edutainment intervention for gender-based violence, sexual and reproductive health, and maternal and child health in rural Senegal: a process evaluation

**DOI:** 10.1186/s12889-022-13570-6

**Published:** 2022-06-10

**Authors:** Agnes Le Port, Moustapha Seye, Jessica Heckert, Amber Peterman, Annick Nganya Tchamwa, Malick Dione, Abdou Salam Fall, Melissa Hidrobo

**Affiliations:** 1 Montpellier Interdisciplinary center on Sustainable Agri-food systems (MoISA), University of Montpellier, CIRAD, CIHEAM-IAMM, INRAE, Institut Agro, IRD, Montpellier, France; 2 Laboratoire des Transformations Économiques et Sociales - Institut Fondamental d’Afrique Noire- Ch.A. Diop, University of Cheikh Anta Diop, Dakar, Senegal; 3 International Food Policy Research Institute, Washington, D.C., USA; 4 University of North Carolina at Chapel Hill, Chapel Hill, USA; 5 International Food Policy Research Institute, Dakar, Senegal

**Keywords:** Edutainment, Process evaluation, Senegal, Gender-based violence, Sexual and reproductive health, Maternal and child health

## Abstract

**Background:**

Edutainment aims to spread educational messages in an entertaining way, and often reaches large audiences. While studies increasingly report the impacts of edutainment interventions, there is limited context-specific evidence on the underlying processes and barriers to effective delivery, especially in rural areas. This article presents results from a process evaluation of a community-based edutainment intervention designed to improve knowledge, attitudes, and practices on gender-based violence (GBV), sexual and reproductive health (SRH), and maternal and child health. The intervention focused on the television series, *C’est la Vie!,* screened through biweekly film clubs in rural Senegal and included post-screening discussions and thematic workshops, meant to reinforce messages, increase knowledge, and change social norms. The objectives of this study were to assess intervention adaptation, implementation fidelity, participants’ responsiveness or engagement, and series appropriateness.

**Methods:**

The intervention was implemented from December 2019 to March 2020 in 120 villages in Kaolack and Kolda regions of Senegal, and targeted adolescent girls and young women aged 14 to 34. The process evaluation was carried out in March 2020 in 14 villages using: i) individual semi-structured interviews with implementers (*n* = 3), village chiefs (*n* = 8), married women (*n* = 9), adolescent girls (n = 8), and men (n = 8); ii) focus groups with men (*n* = 7, 29 participants) and women (*n* = 10, 100 participants); and iii) observations of screening sessions (*n* = 4) and post-screening discussions (*n* = 2). Data were analyzed using thematic and content analysis.

**Results:**

The results highlight that adaptation of the intervention helped reach the target population and improved participant attendance, but might have compromised fidelity to original design, as intervention components were shortened and modified for rural delivery and some facilitators made ad hoc modifications. The screenings coverage and frequency were adequate; however, their duration was shortened due to COVID-19 restrictions in Senegal. Participant responsiveness was excellent, as was the series appropriateness for most topics, including GBV. SRH remains a sensitive topic for youth, especially when the film clubs included non-peers, such as slightly older women.

**Conclusions:**

This study showed that using film clubs to deliver sensitive edutainment content in rural areas is feasible and has potential for scale-up.

**Supplementary Information:**

The online version contains supplementary material available at 10.1186/s12889-022-13570-6.

## Background

Gender-based violence (GBV), sexual and reproductive health (SRH), and maternal and child health (MCH)^1^ remain persistent concerns throughout sub-Saharan Africa, especially in rural areas where many key indicators lag behind urban areas. In 2017, sub-Saharan Africa alone accounted for roughly 66% of the estimated global maternal deaths, with a lifetime risk for maternal death of 1 out of 38 [1]. In West Africa, where this study takes place, regional estimates from 2015 to 2020 found that 32% of women received no antenatal care and only 53% had complete antenatal coverage [2]. Twenty eight percent (28%) of women used contraceptives, with 17% in West Africa and 24% in sub-Saharan Africa having unmet need for contraceptives [3]. Additionally, 33% of ever partnered women aged 15 to 49 in sub-Saharan Africa report having experienced intimate partner violence (IPV) during their lifetime and 20% during the past 12 months [4]. In West Africa, IPV prevalence is even higher with 44% of women reporting IPV in all forms—physical, sexual or emotional—during their lifetime, and 32% reporting IPV during the past 12 month [5].

Social and behavior change communications (SBCC) initiatives are an important component of comprehensive strategies to improve GBV, SRH, and MCH outcomes. SBCC has been widely used in low- and middle-income countries to increase knowledge; change attitudes, beliefs, and norms; and ultimately improve behaviors and practices across multiple health outcomes [6–10]. It is often adapted to specific local contexts and delivered through intrapersonal communication at the individual or group level, in health facilities, or via community outreach. However, the need to increase the reach and intensity of exposure among target populations led policy makers to look for alternative delivery options such as mass media (television and radio) [11]. While radio is widely used in sub-Saharan Africa, access to television varies, but is rapidly expanding. In 2012, 53% of Senegalese households owned a television, which rose to 62% in 2019 (86% in urban area vs 38% in rural area). According to the 2019 Demographic and Health Survey, approximately 63% of women aged 15–49 years old in Senegal watched television at least once a week [12]. The increasing rate of mobile phone driving interconectivity indicates that sub-Saharan Africa is undergoing a digital revolution [13].

Edutainment was defined in 2009 by Wang and Singhal as a “*theory-based communication strategy for purposefully embedding educational and social issues in the creation, production, processing, and dissemination process of an entertainment program, in order to achieve desired individual, community, institutional, and societal changes among the intended media user populations*.” [14, 15]. A multitude of theoretical approaches exist in modern edutainment, with a review of 126 manuscripts published between 2005 and 2016 identifying 56 separate explicit theories operating at the individual and social levels (or a combination of the two) [16]. The most common theoretical approach identified was the Sabido method developed in the 1970’s [17] with the first “telenovelas” produced in Latin America. The Sabido method is based on Bandura’s (2004) social cognitive theory and has been promoted and tested in different settings [18]. Edutainment capitalizes on how viewers or listeners relate to the characters and engage in the story lines, which may be a particularly effective strategy in targeting adolescents and young adults who have relatively high levels of media exposure [19–23] and for whom television is often considered a “super peer” that defines their norms and influences their behavior [24].

Despite edutainment’s wide use and an increasing number of studies that evaluate the impact of edutainment interventions [25–27], there is limited evidence on the processes that underlie effective edutainment delivery and the potential barriers across a wide variety of contexts [28, 29]. In addition, evaluations to date have primarily focused on urban areas, with comparatively more educated and wealthy populations. We conducted a process evaluation to assess the implementation of an edutainment television series, *C’est la vie!,* in rural Senegal, delivered via film clubs and paired with post-screening discussions and workshops. The series revolves around daily life in a Senegalese health clinic and focuses on themes related to GBV, SRH and MCH. The intervention was targeted to adolescent girls and young women. Understanding implementation barriers related to both infrastructure and social norms in a remote and socially conservative environment are important for interpreting impacts, replicating, and generalizing to other settings. The primary research questions of this process evaluation were:

1. To what extent was the delivery of the edutainment intervention adequately adapted for rural settings and for reaching the target population (**intervention adaptation**)?
2. Was the intervention delivered as planned in terms of adherence—content, coverage, frequency, duration—and quality (**implementation fidelity**)?
3. Were participants satisfied with and engaged in the activities, and to what extent did they like the intervention (**responsiveness to the intervention**)?
4. To what extend was the intervention appropriate for the target rural population (**appropriate content**)?

The process evaluation also sought to understand the contextual factors, especially the social norms surrounding key outcome areas (GBV, SRH and MCH) and describe the impact pathways through which the delivery of *C’est la Vie!* may lead to changes in key outcomes. These components are outside the focus of this paper, however additional details are included in the discussion of results where relevant. Our paper contributes to the literature on the effective delivery of edutainment programs by focusing on a comprehensive television series with complementary education activities, in a rural and conservative context in West Africa.

## Methods

### Development and production of C’est la Vie!

*C’est la vie!* was created in 2016 by the *Reseau Africain d’Education à la Santé* (RAES), a Senegalese NGO and produced by Keewu Production. The project began with a 2013 workshop in Dakar, which gathered African storytellers and production teams with the goal of training them on the Sabido method, to produce a Pan African edutainment television. Characters were based on extensive formative research and content formulated around key messages and good practices to promote in each episode. Storytellers wrote scripts that were validated and approved prior to production by a Review Board, which included thematic experts.^2^ By 2021, RAES had produced three seasons of *C’est la Vie!* in French and dubbed it into five local languages, in addition to creating radio adaptations, mini-series for viewing on the internet, social media materials, and community activities.

The RAES SBCC team also developed pedagogical kits, consisting of a post-screening discussion guide covering specific themes presented in Seasons 1 (26 episodes) and 2 (36 episodes), and 7 thematic workshop kits. The post-screening discussion guide is intended to support trained facilitators in leading a discussion after each viewing and consists of principal themes (usually 2 themes per episode) and questions for each episode. The duration of the discussion per episode is expected to last about an hour. The 7 thematic workshop kits cover 5 themes (2 themes are repeated) and include guides, games, posters, and video extracts from *C’est la vie!* in French*.* The workshops are intended for groups of up to 15 participants, lasting half a day with activities including: plot analysis (1 h); discussion of the relation between the series and the participant’s life (30 min); discussions around false (incorrect) beliefs (30 min); and situation analysis and decision-making (30 min).

### Study intervention

This process evaluation documents and analyzes the rural delivery of Season 1 of *C’est la vie!* through local film clubs implemented by the Senegalese NGO MobiCiné, which offers “the mobile cinema near home.” Film clubs took place at community primary schools and target participants were females aged 14 to 34 years. Thirty-four females per village were invited to participate and received two ticket invitations at the start of the intervention, one for themselves and one for a guest. Half the participants in each village received a verbal suggestion to bring a male guest and the other half a female guest (however there were no consequences if the women did not bring the suggested guest).^3^ The purpose of allowing a guest invitation was to both increase the reach of the intervention, as well as incentivize attendance by allowing participants to watch the series with friends and family. Biweekly screenings of three episodes each (26 total) occurred in the local language (Wolof or Pular) and lasted approximately 75 min. See Additional file 1 for episode themes. Discussions after each screening lasting 30 min and 4 monthly thematic workshops lasting 1.5 hs were organized in some villages using the pedagogical kit developed by RAES.

Twelve female facilitators and 12 male technicians were hired by MobiCiné. Facilitators and technicians were first trained over 2 days on the use of the audio-visual equipment (mainly for the technician), logistics, field management, appropriate behavior, and delivering entertainment interventions. RAES conducted an additional one-week training on the delivery of the pedagogical kits, based on practical role-playing sessions and focusing on how to prompt discussion among participants, and how to manage sensitive topics. Each facilitator-technician team was responsible for 10 villages, under the supervision of a regional coordinator.

### Study setting and design

The study was conducted in two regions of Senegal—Kaolack and Kolda—where the main languages are Wolof and Pular, respectively. The process evaluation was embedded in a randomized control trial (RCT), which aimed to quantify the impact of the intervention on knowledge, attitudes and behaviors. The RCT included 120 rural villages assigned before the start of the intervention to one of three arms:

- Arm 1 or CLV: *C’est la vie!* Season 1
- Arm 2 or CLV + kit: Same as Arm 1, plus post-screening discussions and workshops.
- Arm 3 or Control: A television series placebo (“Golden”, not related to the outcomes of interest)

The criteria for village selection were having 1) a primary school, 2) fewer than 500 households, and 3) at least 34 women living within 2 km of the school. Women were eligible if they 1) met the age criteria (14–34 years old), 2) spoke and understood Wolof or Pular, and 3) lived within a 2 km radius of the primary school. In villages with more than 34 eligible females, program participants were randomly selected from all eligible individuals in a village. The baseline survey for the RCT was conducted from October through December 2019. Implementation of film clubs and pedagogical kits was planned over a five-month period, from December 2019 to April 2020. The process evaluation was carried out in March 2020.

### Pilot and inception phase

Prior to the implementation of the intervention, a pilot study was conducted in six villages (two in each region) in April 2019 with the objective of determining the most effective way to implement the study and assess study feasibility and acceptability. In particular, the pilot was meant to determine: 1) optimal location and group size; 2) best days, time, and duration for the screening; 3) optimal way to invite women, and potential to include men, or couples; 4) expected attendance in film clubs; 5) an incentive system to ensure high participation for 6 months; 6) a monitoring system for screenings and discussions. We conducted qualitative exit focus groups (FG) after screenings. Results from the pilot were then presented at an inception workshop where decisions were made based on both pilot results and logistical constraints.

To understand pre-intervention adaptations, we detail the decisions made after we conducted the pilot study. In terms of location, it was determined that school classrooms were the best option, especially compared to screening outdoors. This limited the size of the group (large groups were too noisy and hard to manage) and ensured quality of viewing (lighting and noise control). Although women preferred screenings on Wednesdays (which coincided with short school days) and Sunday evenings, the exact time and day of the screening in each village was determined by the facilitator’s schedule and need to conduct the screening after school hours but before nightfall. Women also preferred weekly screenings of *C’est la Vie!,* and while some preferred only watching 1 or 2 episodes per screening (25–50 min), others thought this was too short. Based on participant preferences and budgetary constraints, it was decided that screenings would occur every other week and 3 episodes would be screened together (75 min), allowing for Season 1 to occur over a 5-month period. While screening 3 episodes seemed to be optimal in terms of women becoming immersed in the plot line and being neither too short nor too long, it also meant that adaptations had to be made to the post-screening discussions, as staying to discuss 6 themes (two themes per episode) over 3 h was not feasible. Instead, two themes were chosen (out of six possible) to be discussed over 30 min, with priority given to one theme (and the other to be covered if time allowed). For the workshops, three themes (out of 5) were selected jointly by the research team and RAES: sexuality (workshop 1), sexual violence (workshop 2), and IPV (workshops 3 and 4) (see Table 1 for a full list of post-screening discussion and workshop themes). Because of the need to keep groups small, it was decided that the first two workshops would invite adolescent participants (aged 14–19 years, including invitations to boys of the same age group as guests), and the IPV workshop would invite participants of any age (and include invitations to their partners where applicable). The IPV workshops were split into two parallel sessions to manage group size. The monthly workshops were reduced from a half day to 90 min for implementation feasibility.

**Table 1:** Themes for post-screening discussions and thematic workshops

Screening session	Episode in the guide (Number)	Theme for the post-screening discussions	Priority	Monthly Thematic Workshop (Number and topic)	Target population
1	3	Child marriage: marital rape and teenage pregnancy	1	1 Sexual health	Adolescent girls and boys (14–19 years old)
2	5	Intimate partner violence	1		
	5	Birth spacing	2		
3	9	Intimate partner violence: lack of freedom and community indifference	1	2 Sexual violence	Adolescent girls and boys (14–19 years old)
4	10	Child, early and forced marriages	1		
	12	Childbirth	2		
5	13	HIV and AIDS	2	3 Intimate partner violence	Females (14–34 years old) and their partners
	14	Family planning and contraception	1		
6	16	Life in a couple	2		
	17	Family planning and contraception	1		
7	19	Women’s rights in the couple	1	4 Intimate partner violence	Females (14–34 years old) and their partners
8	24	Pregnancy follow-up	1		
	24	Childbirth	2		
9	25	Female genital mutilation/cutting	1		

*Notes:* Second priority themes were not always covered if time did not allow. The intimate partner violence (IPV) workshop was split into two groups to keep the group size small (approximately 17 participants and their partners). It could not be implemented due to the COVID-19 disruption

### Authorizations and invitation system

The pilot highlighted the need to have village chiefs’ support to incentivize community participation and communicate logistics. Thus, village chiefs were engaged early in the process. In three villages in Kolda, chiefs denied their approval, due to conservative religious views. These villages were dropped from the study prior to implementation. Participants in each village were identified from a census prior to the impact evaluation baseline survey. Facilitators invited selected participants mainly by phone or walking door-to-door. In some village’s facilitators rented bicycles or involved community health volunteers (CHVs) in identifying target females. Some ticket distributions were organized in the village chief’s house or at schools. Facilitators mentioned that invitations were presented as a privilege, to incentivize women to participate.

### Data collection and sampling

Data to inform our research objectives was collected from a variety of actors and sources (Table 2). The multiple methods included: pedagogical kit review; monitoring data; structured observations at film clubs; sex-disaggregated FGs; and semi-structured interviews (SSI) with village leaders, CHVs, female beneficiaries (adolescent girls and married women), husbands and partners of beneficiary women, and MobiCiné facilitators.

**Table 2:** Objectives and methods used to assess intervention adaptations, implementation fidelity, participant’s responsiveness and appropriateness

Study domain	Specific objectives	Research methods	Themes/Topics investigated
**Intervention adaptations**	• To document the program adaptations for delivery in rural areas (proactive adaptations) • To document the barriers and facilitating factors encountered by facilitators • To document the local adaptations for delivery made by facilitators (reactive adaptations)	• Pedagogical kit review • SSIs with facilitators	• Facilitator selection and training • Procedures and set-up for film clubs • Management and delivery of screening sessions, post-screening discussions, and workshops • Perceived barriers and facilitators to the intervention delivery • Opinions on film clubs and factors that influence its success or failures
**Implementation fidelity: adherence** (content, coverage, frequency, duration) and **quality of delivery**	• To determine content, coverage, frequency, duration, and quality • To identify the barriers and facilitating factors encountered by participants for attending film clubs and participating; attending and active discussion	• Monitoring data • SSIs with facilitators • Structured observations of screening sessions and post-screening discussions • SSIs with women • SSIs with men • FGs with women • FGs with men	• Management and delivery for screening sessions, post-screening discussions, and workshops • Beneficiaries’ experience with the film clubs: Screenings, post-screening discussions, and workshops • Invited guests (who was invited and why) • Perceived barriers and facilitators to participation, attendance, and active discussion
**Participant responsiveness**	• To describe participants satisfaction/ responsiveness to the activities delivered	• SSIs with women • FGs with women • SSIs with facilitators	• Opinion on the series - Characters liked/disliked - Scenes liked/disliked • Satisfaction with the activities
**Appropriateness for rural populations**	• To determine how beneficiaries perceive the delivery of sensitive topics related to GBV, and SRH	• SSIs with women • FGs with women • SSIs with facilitators	• Sensitive topics - In the series - During discussions

Note: Implementation fidelity according to Carroll et al. [30], intervention adaptation to Stirman et al. [31] and series appropriateness to Proctor et al. [32]. Abbreviations: *GBV* gender-based violence, *SRH* sexual and reproductive health, *SSIs* Semi-Structured Interviews.

The sample is described in Table 3. From each study region, three villages each were selected from the CLV and CLV + kit arms and one village from the control arm for a total of 14 villages. We collected data in the control arm, which had film clubs similar to the CLV arm, but with a placebo film, to see if there were variations in program implementation that could explain differences in program impact, beyond the content of the series. The three villages in the CLV and CLV + kit arms were purposively selected to include one low-attendance village (< 60% of invited females attended the two first screenings) and two high-attendance villages (> 60% attended) per arm in each region. Within each village, individual participants were identified using purposive sampling. Due to ethical concerns, interviews were not conducted with men (husbands or partners of participants), who were from the same households or villages as the female participants. Participants were invited by phone ahead of the data collection team’s arrival to help ensure participation of the target individuals.

**Table 3:** Sampling for data collection

Method	Total planned	Planned in arm 1: CLV	Planned in arm 2: CLV + Kit	Planned in arm 3: Control (placebo series)	Total conducted	Gap primarily due to COVID-19 disruption
Villages	14	2 Low and 4 High^a^	2 Low and 4 High^a^	2	14	
Screening observations	8	4	4	–	4	−4 in Kaolack
Post screening discussion observations	4	NA	4	NA	2	−2 in Kaolack
Thematic workshops observations	2	NA	2	NA	0	−1 in each region
FG women	10	4	4	2	10	
FG men	8	4	4	–	7	−1 in Kaolack
SSI married women	8	4	4	–	9	+ 1 in Kaolack
SSI adolescent girls	8	4	4	–	8	
SSI men	8	4	4	–	8	
SSI village chiefs	8	0	4	–	8	
SSI community health volunteers	4	0	0	–	4	
SSI facilitators^b^	4	–	–	–	3	−1 in Kaolack

^a^Low and high refer to attendance levels— < 60% or > 60% of invited women attended the two first screenings, respectively

^b^All facilitators conducted activities in all three arms, within one region

*Abbreviations:FG* Focus Groups, *SSI* Semi-Structured Interviews

The data collection team consisted of ten interviewers (3 men and 7 women) from the *Laboratoire de Recherche sur les Transformations Economiques et Sociales* (LARTES). All interviewers spoke Wolof, and three spoke Pular. They received a one-week training to understand the data collection instruments, harmonize translations, and conduct a one-day pilot. The training also addressed ethics and management of sensitive topics such as GBV. All interviews and FGs were conducted by same-sex interviewers, audio-recorded, transcribed and translated from Pular or Wolof into French, by the interviewers. Interviewers took detailed notes during the film club observations and wrote narrative summaries immediately following the observations. Structured observations of film club screenings and post-screening discussions documented the specific course of events; the observations were not guided or directed by the observer.

Data collection was interrupted by the COVID-19 outbreak, and the difference between the planned and actual samples is shown in Table 3. The majority of interviews and FGs were completed, but we were not able to complete structured observations of screenings and post-screening discussions in Kaolack, or structured observations of any of the workshops. On average FGs with women had 10 participants, ranging from 4 to 19 participants. FGs with men had fewer participants, as men were often absent from villages or occupied (average of 4 participants, ranging from 3 to 7). Despite these limitations, we believe the data collected were sufficient to address the primary research questions.

### Data analysis

Data analysis was based on thematic analysis. First interviews and FG transcripts were coded. A priori codes derived from the interview guides were developed [33]. Additional (a posteriori) codes were added as new themes emerged. The list of codes was reviewed and agreed upon by four of the authors; two of whom coded the interviews and FGs. All coding was reviewed by the first author and discrepancies settled by mutual agreement. The data were analyzed using qualitative content analysis [34]. Film club observations were not coded, as only four observations were completed, but provided useful information which we triangulated with other findings. Coding and analysis were conducted in NVivo 13 (QSR International).

### Ethics approval

Written informed consent was obtained from all participants. For minors, written assent was obtained, along with written informed consent from the legal guardian. Village chiefs who were interviewed received a bag of tea as a token gift. Other study participants were not compensated. Due to sensitive questions around GBV, women interviewees were offered de-identified referral information for social and legal region-specific services, as well as the number to a toll-free national GBV helpline. For women requesting immediate assistance, interviewers collected referral information and liaised with services who provided direct follow-up. The study was approved by the *Comité National d’Ethique pour la Recherche en Santé* in Senegal (#00000929 MSAS/DPRS/DR) and by the Institutional Review Board of the International Food Policy Research Institute (IFPRI) (#00007490).

## Results

To assess implementation fidelity, we examine screening sessions, post-screening discussions, and workshops, including their delivery and uptake. Barriers and facilitating factors for delivery, participation, attendance, and active discussion were identified throughout, and when made, post-implementation adaptations to overcome those barriers are described. Participants’ responsiveness and appropriateness of the intervention for rural populations was explored by documenting opinions and satisfaction around activities and sensitive topics.

### Implementation fidelity

#### Screenings

Facilitators informed women of each screening by calling them directly or calling the village chiefs or CHVs, who relayed the information, especially to those more difficult to contact. In villages where mobile connectivity was weak, students usually returned from school and informed their mothers that MobiCiné had arrived. Facilitators indicated film clubs started between 2:30 and 5:00 PM., depending on women’s activities and on MobiCine’s arrival time. Sometimes, schedules could be problematic: the screenings were too late for women who lived far from the film clubs, leading facilitators to modify screening times, or in a few villages rent horse-drawn carts, to ensure that women could return home during daylight hours. Dispersion and distance between households and the primary schools was also a problem when facilitators searched for absent women.

The four structured observations conducted in Kolda indicated that classrooms were of moderate size and ended up crowded with insufficient seating. The densely packed rooms and heat meant that the space easily became overheated in the hotter months. In addition, children and adolescent boys would watch the series through windows or doors. Both the heat and noisy environment could have lowered screening quality. However, women interviewed were satisfied about the quality of the screening sound and images. No technical issues were reported. The duration (75 min) was considered acceptable by most women.

The monitoring data reveals that the first three screening sessions were implemented in more than 90% of the villages and the fourth in approximately 80% (Table 4). Sessions 5 and 6 were in progress when the COVID-19 outbreak occurred with session 6 only implemented in one-third of the villages. Nine sessions were planned to view all of Season 1, however sessions 7–9 were never implemented due to COVID-19. Approximately 60 to 70% of target females attended the first two sessions in Kolda and around 50% continued for the next four sessions. Attendance in Kaolack reached 58% at session two and decreased slightly to around 45% in the remaining sessions. Invited guests reached a maximum of 43% attendance.

**Table 4:** Film club implementation and attendance

	Kaolack (*N* = 60)				Kolda (*N* = 57)			
	Villages with screenings completed		Attendance of target females^a^	Attendance of invited guests^a^	Villages with screenings completed		Attendance of target females^a^	Attendance of invited guests^a^
	%	N	%	%	%	N	%	%
Session 1	100	60	54	38	100	57	70	41
Session 2	100	60	58	43	98	56	65	29
Session 3	97	58	48	31	93	53	56	23
Session 4	82	49	41	35	79	45	55	20
Session 5	63	38	45	37	40	23	46	21
Session 6	37	22	45	37	30	17	49	23

*Note:* Statistics are aggregates for all study arms (CLV, CLV + kit and the control placebo). Data source are monitoring data recorded by MobiCiné (implementing NGO)

^a^In villages with screenings completed

At the individual level, Table 5 shows that 85 to 88% of the targeted participants attended at least one screening, with participants attending on average 2.3 to 2.6 screenings out of an average of 4 screenings implemented in the villages. Several reasons for not attending film clubs were cited by participants and facilitators, such as not being aware of date and time, busy with housework (fetching water or cooking), traveling (to markets or religious events), and illness. A few beneficiaries mentioned lack of motivation, due to the heat in Kolda, already having seen the series, and wanting a financial incentive. Facilitators’ introduction and integration into the village, so the facilitators were *“not seen as a stranger*”, was essential for women. The appeal of the series was also important to encourage women to return to the screenings. As one facilitator explained, *“For the women, when they watched the first screening of the film, they became interested in it and were motivated to continue to come and watch the rest of the series” (Facilitator 3, Kolda).* Permission from husbands, mothers-in-law, parents, or other family members was almost always a prerequisite for attending film clubs. Authorizations could also support or incentivize women to participate, as stated by a respondent, *“All men are happy with your program. Personally, it is my husband who urges me to come and watch the series” (Female FG10, CLV, Kolda)*.

**Table 5:** Adolescent girls’ and young women’s attendance at film clubs by study arm

	Full sample	Arm 1: CLV	Arm 2: CLV + Kit	Arm 3: Control
Average number of screenings implemented in the villages (mean, SD)	4.3 (1.3)	4.2 (1.3)	4.5 (1.3)	4.3 (1.2)
Total females aged 14–34 years targeted for the intervention (N)	3965	1288	1359	1318
**Screenings**				
Attended screening sessions at least once (%)	87.2	88.2	87.8	85.8
Attended all screening sessions (%)	31.9	33.0	29.8	33.0
Average number of screenings attended (mean, SD)^a^	2.3 (1.5)	2.3 (1.4)	2.6 (1.6)	2.3 (1.5)
**Post-screening discussions**				
Attended post-screening discussions at least once (%)	77.4	NA	77.4	NA
Attended all post-screening discussions sessions (%)	48.1	NA	48.1	NA
Average number of post-screening discussions attended (mean, SD)	2.1 (1.6)	NA	2.1 (1.6)	NA
Invited someone at least once (%)	63.0	58.1	63.9	67.7
Invited a male guest at least once (%)	24.0	25.3	26.3	20.4
**Thematic workshops**				
Adolescent girls aged 14–19 years (N)	NR	NA	521	NA
Adolescent girls attended workshop at least once (%)	NR	NA	22.0	NA
Adolescent girls attended 2 workshops (%)	NR	NA	3.2	NA

*Notes:* Data source are monitoring data recorded by MobiCiné (implementing NGO). Adolescent attendance is only reported for Arm 2 (CLV + Kit) as separate workshops were organized for adolescents only in this study arm. Abbreviation: *CLV* C’est la Vie!, *SD* Standard Deviation, *NA* Non Applicable, *NR* Non Reported

Nearly all participants interviewed who took a guest to the screening brought a female friend or neighbor, co-wife, sister-in-law, or other female guest. Adolescent girls often chose to invite their mother, sister, sister-in-law, aunt, or friend, because of their “*trust”, “availability*”, or being *“of the same generation”.* Participants were sometimes accompanied by their parents at the start of the intervention, to confirm the suitability of the content, as mentioned by an adolescent whose mother would tell her about the “*pros and cons*” and the “*dangers*” of attending the screenings. In one village, two married women came with their sons. Only one woman participating in a FG indicated that her husband accompanied her regularly, explaining, “*He likes the series a lot because he saw the importance*” *(Female FG9, CLV + kit, Kolda).* Monitoring data confirms that among all females who participated, while 63% ever attended with a guest, only 24% ever attended with a male guest.

#### Post screening discussions

In the CLV + kit arm, facilitators led discussions following the screenings. They were described as follows, *“We show video extracts that they have seen on the show. We ask them what they thought about what they watched on the show. We discuss it, and it helps them understand” (Facilitator 1, Kaolack).* Facilitators self-described role was to “*give women a voice to allow each one to express themselves,*” “*give each participant the floor and let them speak,*” and to “*reorientate the discussion if necessary.*” Facilitators felt the discussion guides, which started off with general questions to ease participants into the discussion, were well adapted to the target population, easy to use, and effective. However, one facilitator indicated she was not using them to maintain spontaneity in the discussions and to avoid seeming like an “expert”, possibly compromising program fidelity if she was not adhering to the pre-agreed themes.

The two observations of post-screening discussions showed some variations in delivery, with durations between 30 and 45 min. In one, the facilitator did not screen an extract and led discussions on contraceptives (16 min), family planning (10 min), and sexually transmitted infections (STIs) (3 min). Only adult women spoke, not adolescents, which may have been due to the topic. In the other, one extract was screened, and the discussions focused on gender roles, family planning, and contraceptive use (15 min each). These variations in sequences of activities, themes, and duration could have compromised implementation fidelity and quality of delivery.

The monitoring data (Table 5) revealed that 77% of the targeted participants in the CLV + kit arm attended at least one post-screening discussion, 48% attended all post-screening discussion sessions, with the average number of sessions attended being 2.1. All participants from the CLV + kit arm interviewed during the process evaluation reported attending the post-screening discussions. They were motivated to attend, because “*the discussions makes sense,*” are “*interesting,*” the organization of the discussions are good, and “*there are things that can be useful.”* Participants indicated that they did not want it to end because they “*feel affected by the things that are done to women.*” The work of the implementers was said to be “*perfect.*”

Facilitators gave more details. The most appealing aspects of the post-screening discussions to participant females were the dialogue and discussion, which were a source of motivation and allowed participants to absorb and internalize messages. Discussions, including the sharing of experiences by respected people (elder women and men that would spontaneously come to the screenings even if not invited) in the village, and the space to ask questions that deepened their comprehension. The ambience and the curiosity with what other women said was also important: *“...you see two people, you ask the question, you see them whisper between themselves. There are others who want to know what the other is going to say. It’s the atmosphere that they like or the content that we’ll bring out” (Facilitator 3, Kolda).*

#### Thematic workshops

At the time of the data collection, only the workshops on sexual health (Workshop 1) and sexual violence (Workshop 2), which targeted adolescent girls, had taken place (only in Arm 2). According to monitoring data, among the adolescent girls targeted, only 22% attended at least one workshop. Workshops were scheduled for the middle or late afternoon, typically on Saturdays and the few adolescents interviewed during the evaluation, who attended a workshop, were satisfied with the day and time it was held. Facilitators had very positive opinions of the workshops, because they felt the activities were results oriented. However, the duration of the workshops was considered too short to complete all the activities.

Facilitators described the two main activities as games: i) situation and decision cards, considered easy to manipulate because of simple messages and ii) Regnier’s abacus,^4^ a tool for expressing opinions on a topic and encouraging discussion of alterative viewpoints. Both activities were seen as effective and fun ways to convey knowledge. Regnier’s abacus was the preferred activity for one of the facilitators who explained it allowed for debate in a fun way, “*as if we were in court. Some say one thing and others say the opposite” (Facilitator 1, Kaolack).* Another facilitator remembered: *“It really opens up the discussion, because they are comfortable. They are so interested. Well, I had to do a workshop for 2 h, they were there, and they wanted to continue the discussion” (Facilitator 3, Kolda).* Adolescent girls also recounted the interactive games and the topics: *“The topic that I liked the most in the discussions was that of violence, because if you are a victim of rape you have to talk to your mother. We discussed it”* (*Female FG7, CLV + kit, Kaolack)*.

Facilitators liked the workshops because they could work with small groups of individuals facing similar issues. A facilitator mentioned increased attendance between the two workshops and enthusiasm among young people for the activities (friends were invited). Some participants offered to conduct the workshops themselves. Teenage girls enjoyed decision cards and roleplaying, “*which allow young people, while playing, to work without realizing it.*” However, one facilitator indicated that the limit of 15 participants per workshop was too small, given the number of people in some villages. This facilitator allowed more than 15 participants, and then faced the problem that tools were developed for a small group.

One barrier to participation was the use of French in the game materials and general literacy levels—particularly in Kolda. Most adolescents could not read the cards in French, thus forcing the facilitator to read them. Additionally, the video clips for the workshops were in French and needed simultaneous translation into Pular or Wolof. Guides for the workshop were considered more adapted to urban youth and those with stronger French fluency, which is uncommon in the study area. Overall, although facilitators had a positive opinion on the workshops, the limited duration of the workshops (1.5 h instead of half a day), the fact that more participants than planned participated in the session and the need for translation could have lowered the quality of workshops implementation.

### Participants’ responsiveness

#### Series content

Participants unanimously expressed their enthusiasm for the series and intervention. They enjoyed the series, because of the novelty and its messages, as access to this type of information is often limited in rural areas. Participants liked the show because it was interesting, important, and it “*raised their awareness*.” The series *“awakens”* them, for example, to discover how society works and encourages reflection; “*We get to know many things that we didn’t know before, we didn’t have this opportunity [ …*.] *through these films we can see that girls are mistreated, threatened and abused in this society... These are real things that happen in life, but we didn’t know because we live in rural areas” (Female FG4, CLV + kit, Kolda)*. The series also enables them to reinforce their knowledge and learn new material. *“It was in the series that I learned that early marriage was not good because it can cause health problems for a child when she gets pregnant. Just like the children that are raped and the young girls that are given away early because of money” (Female FG1, CLV, Kaolack)*. Finally, the series helped them to make the right choices and enabled them to advise others: “*For example, for us married women, we cannot go back but we can still protect our children against certain practices such as child marriage” (Female FG10, CLV, Kolda)*.

#### Favorite and most-hated characters from the series

Participants cited a wide range of characters from the series as favorite or hated, thus showing their immersion and responsiveness to the series. They knew the names and plot lines of the characters, and frequently shared passages from the series to justify their choices. The protagonist midwife was most commonly identified as the favorite character, because she is “*nice*,” enjoys her job, and looks after and treats her patients well: “*It’s because of her that I love the film*.” Others included victims of mother-in-law abuse and IPV, because of their strength and courage in dealing with the abuse. The most-hated characters were an evil-natured midwife who steals patients’ money, abuses and neglects them, has a bad attitude and lacks a spirit of collaboration, and the abusive mother-in-law because she interferes in the life of couples and abuses her daughter-in-law (verbal abuse, denigration, makes her do all the housework and plots to find a second wife for her son). The violent husband was also hated.

#### Themes and plots that were liked and disliked

When asked about what themes they liked, a large majority of participants in both regions simply answered that they liked everything. Women’s health and the focus on the daily lives of women were mentioned several times: *“What I like the most about the show is that we learned a lot of things there like how to run the household and a lot of other things in our daily life.” (Female FG10, CLV, Kolda).* The scene where the midwife helped a young woman give birth, was often cited as a happy moment, creating positive emotions.

There were many unpleasant plot lines that touched on GBV and evoked strong emotions. Those related to IPV left their mark: “*A part that I didn’t like, for example, was when the husband hit his wife until he broke her arm. You really know that what he did to his wife is not good at all. He stole her gold box to sell it. And I don’t know if he bought it or not, but whatever the problem, he shouldn’t hit her” (Female FG1, CLV, Kaolack*). Scenes of violence by the mother-in-law and scenes about marital rape in the context of child marriage were also cited: *“What I didn’t like the most was the death of the girl. Indeed, she died because she had an old (er) husband who forced her to sleep with him “(Female FG3, CLV + kit, Kaolack).*

### Appropriateness

#### Reaching target audiences

According to facilitators, the series was well adapted to adolescent girls in the target communities, who were often unoccupied outside of school, and to pregnant or lactating women interested in SRH or MCH. A CHV indicated that in her village, younger beneficiaries were more interested in the topic of early pregnancies, while older ones were interested in IPV. However, they reported challenges in involving men. Although men were not the main target, their participation as guests was encouraged for half of the female participants. The intervention design relied on women being responsible for inviting and motivating their husband or male family member or friend. As one facilitator explained, *“For the men to come, it is their wives who can motivate them. It is up to them to explain the interest because alone there will be no change but if they try to convince their husbands and explain the interest they will come” (Facilitator 3, Kolda).* Many men also claimed that they were not informed by their wives**.** Other men felt their wives discouraged them from attending: *“My wife never invited me to go to the film club [...]. I did go for the first screening, but when I was told that they only needed women I turned back” (Male FG4, CLV + kit, Kolda).* Some husbands came once and never returned, considering the series “*for women*”, as indicated by a facilitator, *“The problem we have is for men … at my first screening I had a room full of men. The men came. There were 11 or 13 of them, but at the end of the first episode they all left, and they told me the same thing: this is for the women, because we only saw secretaries.*^5^*In fact, we very rarely saw men in the film” (Facilitator 2, Kolda).*

Most women were in favor of being able to watch the series without men present. *“I think it’s better if we watch the series alone as women and then talk about it”* (*Female FG7, CLV + kit, Kaolack*). A woman said that she went with her son but “*kids can’t keep secrets.*” An adolescent girl explained that there should be no embarrassment for young girls to watch the series with boys, but that she felt ashamed and also preferred to watch without parents: *“I prefer that young people watch alone without their parents. If it’s with women it’s not a problem, but with men you will feel ashamed” (Female FG7, CLV + kit, Kaolack).* Additionally, young unmarried participants who have boyfriends would not dare invite them and risk them meeting their mothers or their mothers’ friends.

#### Sensitive topics

One main concern about the use of the series was related to the sensitivity of topics explored, especially for rural, more conservative population. According to the facilitators, sensitive issues could undermine participation in the discussions and workshops. They mentioned the following sensitive topics: (i) sexual health and behavior, (ii) use of family planning by unmarried women, and (iii) IPV.

Sex was a sensitive subject, especially when it was approached from the perspective of women’s independence and ability to control their sexuality and choice of sexual partners. This narrative shocked viewers, because it goes against social norms that forbid premarital sex, as explained by a facilitator: *“So you see that some topics are not adapted to the realities of the region. Today, we talked about the sexuality of an independent woman, but even your boyfriend is not allowed to approach you. A woman should not have sex before marriage, you see” (Facilitator 1, Kaolack).* Family planning was also sensitive, as it is normally intended to help married women space births, not for unmarried women to prevent pregnancy. According to a facilitator, family planning has only recently been introduced in some villages, hence interest in the topic; “*They like to discuss early marriage, but also family planning. Women have not been practicing planning for a long time here, but they discuss it very often” (Facilitator 1, Kaolack).* IPV did not generate much discussion, according to a facilitator,: *“On intimate partner violence, they are not very comfortable when we talk about these subjects. Because for them domestic violence is normal. Because they say that it is between you and your husband … “ (Facilitator 3, Kolda)****.***

Most female participants interviewed felt comfortable opening up to the facilitator and participating in post-screening discussions but mentioned that others might feel uncomfortable or be afraid to speak in public. For example, lack of confidentiality and fear of judgement in the group setting prevented some from expressing their opinions: *“The difficulties are the fact that a subject is really taboo and [...] that everyone is silent, because they say to themselves, if I say a case in relation to such a subject, people will think that it is because I am doing it, but with a little reassurance they open up. And if only one person starts to speak, everyone speaks” (Facilitator 2, Kolda).* In some villages, the presence of men, religious authorities, elders and mothers-in-law prevented women from expressing themselves during post-screening discussions, as explained by a woman in Kolda*: “Some would feel comfortable, others would feel very embarrassed to express themselves. It is the presence of men that makes them feel uncomfortable. If women were alone, they would not feel ashamed” (Female FG3, CLV + kit, Kaolack)*.

Facilitators found adolescents were reserved when discussing sex in the presence of older women, but expressed themselves more freely during the adolescent-only workshops. Another facilitator, however, explained post-screening discussions to be a unique opportunity to discuss sensitive subjects in a multigenerational environment: *“There are moments when almost all my targets are nieces of the aunts. During these moments, they have the opportunity to discuss very delicate and taboo subjects between parents and children, and there the mother can say what she thinks, as well as the child. It is like an intergenerational dialogue where women and mothers talk and each one could bring out their feelings, thoughts and worries” (Facilitator 2, Kolda).* During workshops, among the 14 to 19 years old adolescent girls, some were already married or pregnant, and therefore “*not comfortable on certain topics, because they have already experienced this and they were a little embarrassed to talk about it” (Facilitator 3, Kolda).*

## Discussion

This study examines the implementation of a rural edutainment series, *C’est la Vie!* through film clubs targeted to adolescent girls and young women in Senegal. Specifically, we address questions on the extent of adequate adaptation for the target audience, implementation fidelity, participant responsiveness, and appropriateness of the intervention. The process evaluation was imbedded in an RCT to better understand the impact pathways and improve implementation to maximize impact. Previous research on television- and filmed-based interventions exists, such as series on maternal and newborn health in rural Ethiopia [35], breastfeeding in peri-urban South Africa [36], and protecting oneself against HIV in urban Nigeria [26]. However, the programming is typically of limited length, focuses on urban areas, and does not benefit from complementary educational activities. Meanwhile the related studies mainly focused on the impacts, rather than assessing implementation. To our knowledge, this study is among the first to document the adaptation and implementation of a television-based edutainment intervention delivered in rural areas.

*Adaptations* to community-based interventions are rarely documented. They can be planned—proactively or reactively—at the pre-implementation stage or during implementation, with varying goals, such as improving intervention feasibility or engagement [31]. In the present study, the *C’est La Vie*! implementation initially used a proactive approach, determined through an implementation pilot. Post-screening discussions and workshops were planned by the research team to be lighter in content with fewer themes than those initially designed. This was done to improve feasibility of implementation within time constraints (and time costs to participants) and to not overload the target population with too much information. Results showed that in addition to these pre-planned adaptations, some ad hoc adaptations were made by facilitators (e.g., not following the pre-agreed list of themes, not showing series extracts, having more than 15 participants for workshops), and could have lowered post-screening discussions and workshops implementation fidelity and impact. However, the facilitators also developed reactive and locally adapted strategies to overcome barriers to attendance and participation, such as finding alternative means to communicate film club schedules and renting horse-drawn carts. While the local translation and adaption of the workshop kits would have further reduced barriers, these were not finalized at the time of implementation and thus not tested nor adapted. Overall, these findings on adaptations are important to consider for replication in other parts of the country or in neighboring countries. While local adaptions are important for reducing barriers to participation, implementers should also consider whether these adaptations will threaten the potential efficacy of the intervention.

Through a detailed analysis of the delivery and uptake of the intervention, we also assessed *implementation fidelity*. This includes intervention adherence (content, coverage, frequency, and duration), and is moderated by a number of factors, including quality of delivery [30]. The content of the program was found to be well-adapted to the target audience, and coverage was high, although, not all women attended each session. We observed some differences in attendance between the two regions with higher overall attendance in Kolda as compared to Kaolack. We hypothesize this could be due to the remoteness of Kolda, where populations have lower exposure to movies and other outside social events, making the intervention more novel. On this, a village chief in Kolda commented with enthusiasm that a film club was a surprise: “*We would never have imagined that this would happen within our walls. It’s a nice surprise for us so [ …] Previously, who would have thought that cinema could reach this far? Nobody! The cinema we only heard about in urban areas*.” (*Chief, CLV, Kolda*).

Take-up and retention, which is a common challenge of television-based edutainment, were in-line or higher in our study compared to other television-based edutainment efforts in Africa. For example, an evaluation of MTV’s *Shuga* had initial take-up rates during a screening session of 38% among a sample of urban Nigerian youth, with approximately 78% of those who initially attended the screening subsequently viewing at least one of the two primary series showings [25]. In addition, among women in Egypt invited to view short videos via Facebook or WhatsApp, approximately 45% visited online platforms, watching between two and three videos [37]. Thus, the approach used for *C’est la Vie*!, with initial attendance rates of 90% and approximately one-third of women attending all screenings can be considered relatively successful compared to other approaches.

Female *participant responsiveness* was high across nearly all intervention components. The television format delivered clear and strong messages that were understood by participants. Moreover, participants liked the series, were engaged, shared information and story lines with family and friends, became aware of important issues, and learned. Women could remember and identify with characters and were transported by subplots, which are important pathways for changing behavior [38, 39]. They also found the post-screening discussions and workshops to be educational and enjoyable.

Considering the *appropriateness* [32] of the series content and intervention activities for the target group, the themes presented in the series were of interest, as they covered a large range of content from adolescent to family perspectives on GBV, SRH, and MCH. These topics were less interesting to men, which reduced men’s participation. In addition, sensitive topics—such as sex—could have undermined participation during post-screening discussions and workshops. The most frequently cited reason in terms of hesitancy to engage in discussions was the heterogeneity of participant groups, which sometimes included men and authority figures (partners, teachers, village chiefs, and elders). Although some participants felt embarrassed or shy during the first post-screening discussions or workshops, facilitators helped them feel reassured, and they appreciated the unique opportunity to discuss taboo topics with elders. A recent study with adolescent girls in Kolda, Senegal, showed how valuable it is to create public space for intergenerational conversation. Using participatory, dialogical approaches on some of the same sensitive topics as in our study (child marriage, early pregnancy, and female genital mutilation/cutting) across generations and between sexes, they showed shifts in gender attitudes and actions over an 18-month period [40]. SBCC interventions must carefully balance the tradeoffs of inclusivity and in particular, the benefit of potentially changing norms at the community level with the cost of potentially stifling free exchange of ideas among those with less power and standing.

Finally, SBCC programs typically delivered in rural areas, even when effective in inducing behavior change, typically rely on one-on-one or group counseling conducted by health care workers or peer counselors [7]. Edutainment-based programs and other approaches that use media as a delivery platform could improve cost-effectiveness compared to more traditional SBCC approaches [9, 41]. Our results show that edutainment is an innovative way of communicating on sensitive topics such as GBV and SRH, especially with adolescents and in rural areas, where there are significant barriers to information dissemination. However, even edutainment interventions such as *C’est la Vie!* need time, repetition, and discussion for messages to be internalized and new social norms and behaviors to be adopted, which may limit their ability to be scaled up. When going to scale, such programs should consider innovative ways to deliver complementary educational materials, such as integrating content of pedagogical kits into existing structures to facilitative expansion (e.g. schools, training, women’s groups etc.).

## Conclusion

Overall, our study indicated that the community-based edutainment intervention was implemented as planned before the COVID-19 disruption, and highlighted an excellent responsiveness to the intervention and appropriateness for adolescent girls and young women. However, the pre- and post-implementation adaptations to the post-screening discussions and workshops highlight the need to focus on how to optimally integrate and find a compromise between the ‘educational’ and ‘entertaining’ components. Nevertheless, we demonstrate that using film clubs to show edutainment content in rural areas is feasible and has the potential for scale up. Future research on edutainment should document intervention adaptation, fidelity, participant engagement and series appropriateness alongside impacts for a more holistic understanding of how to improve gender norms and outcomes in rural settings.

## Supplementary Information

**Additional file 1.**

## Data Availability

The datasets used and/or analyzed during the current study are available from the corresponding author on reasonable request.

## Abbreviations

CHV: Community health volunteer
FG: Focus groups
GBV: Gender-based violence
IPV: Intimate partner violence
MCH: Maternal and child health
SBCC: Social and behavior change communication
SRH: Sexual and reproductive health

## Footnotes

1 While various definitions exist, we classify outcomes of interest in this study as follows: 1) GBV refers to harmful acts, including psychological, physical or sexual harm, inflicted on an individual based on their gender, including key thematic areas of intimate partner violence, early and forced child marriage and female genital cutting/mutilation; 2) SRH refers to a state of complete physical, mental and social well-being related to the reproductive system, with key thematic areas examined in this study including early pregnancy, contraceptive use and family planning, sexually transmitted infections and unsafe abortion; and 3) MCH refers to health and wellbeing of women and infants during pregnancy, childbirth and post-partum, including key thematic areas of pre- and post-natal care, facility birth and infant and young child feeding. While some conceptualizations nest components of GBV and MCH within SRH as a broad umbrella, we include them as separate themes following our conceptual framework and pre-analysis plan for the impact evaluation.

2 Experts were from the United Nation Population Fund (UNFPA), United Nations Entity for Gender Equality and the Empowerment of Women (UN Women), United Nations Children’s Fund (UNICEF), and the World Health Organization (WHO).

3 The soft nudge to invite a male or female guest was randomized across all target women purposefully as part of the larger quantitative evaluation, with the objective of assessing if impacts varied by whether men were more (less) engaged in the film clubs.

4 Visual decision-making tool to facilitate collective bargaining. A series of statements is proposed and each participant responds to each statement with a color code, according to his level of agreement. This opens the discussions.

5 This was likely a disdainful comment, as the first episode takes place in the maternity hall, it is likely that the midwifes were mistaken for or framed as “secretaries” at the health facility reception desk.
