# Supplementary material for: A community edutainment intervention for gender-based violence, sexual and reproductive health, and maternal and child health in rural Senegal: a process evaluation
Source: BMC Public Health. 2022 Jun 10;22:1165. doi: 10.1186/s12889-022-13570-6 (PMC9185706; doi:10.1186/s12889-022-13570-6)
Supplement: Supplementary file 1 — Additional file 1. [file 12889_2022_13570_MOESM1_ESM.docx]

**Additional file 1: Themes developed in the C’est la Vie! series, season 1,** **episodes (1 – 26)**

| **Episode** | **Themes** |
| --- | --- |
| 1 : *La nouvelle* [The newcomer] | - Health seeking behavior - Family violence (among co-wives and perpetrated by mothers-in-law) - Work-place violence |
| 2 : *Pour une cuisse de poulet*  [All for a chicken thigh] | - Family violence (among co-wives) - Work-place violence, corruption - IPV - Gender equity (care work) |
| 3 : *Trop c’est trop*  [Enough is enough] | - Family violence (perpetrated by mother-in-law) - Work-place violence - IPV - Child marriage, marital rape |
| 4 : *Pour un bouquet de fleurs*  [For a bouquet of flowers] | - Family violence (perpetrated by mother-in-law) - IPV, reporting to law enforcement - Work-place violence |
| 5 : *Révélations*  [Revelations] | - Family violence (perpetrated by mother-in-law) - IPV - Family planning, birth spacing - Work-place violence |
| 6: *La première fois*  [The first time] | - Family violence (perpetrated by mother-in-law) - Child marriage, marital rape - Dangers associated with abortion |
| 7 : *A qui la faute?*  [Whose fault is it?] | - Family violence (perpetrated by mother-in-law) - Child marriage, marital rape - Dangers associated with traditional medicine, black market drugs |
| 8 : *Prise de conscience*  [Awareness-raising] | - Dangers associated with traditional medicine - IPV - Work-place violence - Sexual assault |
| 9 : *Evasions*  [Escape] | - Infidelity - IPV - Pregnancy complications |
| 10 : *Les amoureux de Ratanga*  [Ratanga’s lovers] | - Infidelity - Forced marriage - Sexual assault - Youth sexual and reproductive health |
| 11 : *Faux-semblants*  [False appearances] | - Sexually transmitted infections - Bride price, marriage institutions - IPV |
| 12 : *Coup monté*  [A set-up] | - Pregnancy and childbirth complications - Work-place violence |
| 13 : *Convictions*  [Convictions] | - Youth sexual and reproductive health, family planning - Sexually transmitted infections |
| 14 : *Des militants à Ratanga*  [The militants of Ratanga] | - Youth sexual and reproductive health, family planning |
| 15 : *L’intrigante*  [The schemer] | - Dangers associated with abortion - Obstetric violence - IPV |
| 16 : *Stratagèmes*  [Tricks] | - Family violence (perpetrated by mother-in-law) - Gender equity (care work) |
| 17 : *Ennemie jurée*  [Sworn enemy] | - Work-place violence - IPV (controlling behaviors around women’s right to work) - Youth sexual and reproductive health, family planning |
| 18 : *Intimidation*  [Intimidation] | - IPV - Maternal and child health, breastfeeding - Youth sexual and reproductive health, family planning |
| 19: *Révoltes*  [Revolts] | - Family planning - Reproductive rights - Work place violence - IPV |
| 20: *Retour forcé*  [Forced return] | - IPV - Gender equity - Intra-personal violence (perpetrated by mother in law) - Child marriage - FGM/C - Antenatal and postnatal care |
| 21: *Trompeuses apparences*  [False appareances] | - Marital conflict - Birth spacing |
| 22: *Seules contre tous*  [Alone against all] | - Last stage of pregnancy and breastfeeding - Child marriage - Intra-personal violence (perpetrated by mother in law) - FGM/C |
| 23: *Un enfant à tout prix*  [A child at all costs] | - Medical confidentiality and the right to information - Illegal abortion - Education |
| 24: *Délivrance*  [Delivery] | - Marital conflict - Youth sexual and reproductive health, family planning - Pregnancy follow-up - Childbirth complications |
| 25: *Vengances*  [Revenge] | - FGM/C (consequences and mortality) |
| 26: *Nouveau depart*  [New start] | - Intra-personal violence (perpetrated by mother in law) - IPV - FGM/C (complications) - Engage for children and women’s rights |

Source: Authors notes and ‘Parlons-en! Un Guide de Discussion Pour Accompagner Chaque Episode Televisé’ (*C’est la vie!*). Abbreviations: FGM/C: Female Genital Mutilation/Cutting; IPV: Intimate Partner Violence.
